# Real-world treatment patterns, outcomes, and healthcare resource use for myelofibrosis in Brazil: Subgroup analysis of the METER study

**DOI:** 10.1016/j.htct.2026.108551

**Published:** 2026-10-27

**Authors:** Fernanda Seguro, Ingrid Pereira, Maria Duran, Nelson Hamerschlak

**Affiliations:** a Hospital das Clínicas da Faculdade de Medicina da Universidade de São Paulo, (HCFMUSP), São Paulo, SP, Brazil; b Instituto do Câncer do Estado de São Paulo (ICESP), São Paulo, SP, Brazil; c AbbVie, Brazil; d AbbVie International, United States of America; e Albert Einstein Israelita Hospital, São Paulo, Brazil

**Keywords:** Primary myelofibrosis, Hydroxyurea, Ruxolitinib, Practice patterns, Physicians, Brazil

## Abstract

**Background:**

The METER study (NCT05444972) described real-world treatment patterns and outcomes for myelofibrosis across 14 countries. Given the potential regional variability, the present analysis evaluates the Brazilian cohort within METER to investigate local treatment approaches and patient results.

**Methods:**

This subanalysis of the METER study included all patients enrolled in Brazil, based on the same eligibility criteria as in the global study. The primary objective was to describe real-world treatment patterns for myelofibrosis.

**Main results:**

Of 50 eligible patients enrolled in Brazil, 37 (74%) presented with primary myelofibrosis. At diagnosis, nine (20.9%) individuals had high-risk and 14 (32.6%) intermediate-2 risk disease, mostly using the Dynamic International Prognostic Scoring System (DIPSS) criteria. Among patients with available data (n = 18), 50% had a high-risk mutation at diagnosis. Hydroxyurea was the most frequent initial therapy (n = 32; 64%), followed by ruxolitinib (n = 12; 24%). A total of 23 patients (46%) had discontinuation or change of initial therapy, with a median overall duration of first-line treatment of 29.9 months (95% confidence interval: 21.5 to not estimable). The median overall survival from index date was 45.5 months (95% confidence interval: 28.5 to not estimable), with 1- and 2-year survival rates of 87.3% and 74.7%, respectively.

**Conclusion:**

The treatment patterns in Brazil reflect insufficient access to novel agents. Whether the shorter observed median overall survival compared with the overall METER study is a consequence of insufficient access to treatment or of different biological features warrants further investigation.

## Introduction

Myelofibrosis is a Philadelphia chromosome-negative myeloproliferative neoplasm characterized by bone marrow fibrosis, constitutional symptoms, hematologic cytopenias, extramedullary hematopoiesis, and hepatosplenomegaly, ultimately leading to reduced survival [1,2]. Potential complications include leukemic transformation, thrombotic and hemorrhagic events, and infections [3]. The therapeutic approach is largely guided by symptoms and disease severity as indicated by risk-stratification systems [1,2,4]. The International Prognostic Scoring System (IPSS), Dynamic IPSS (DIPSS), and DIPSS Plus (DIPSS+) categorize myelofibrosis into low, intermediate-1, intermediate-2, and high-risk disease based on clinical and laboratory criteria [5]. Mutation-enhanced IPSS (MIPSS) further incorporates molecular parameters [5]. While asymptomatic patients typically undergo active surveillance [1,4], symptomatic individuals are managed based on their risk stratification, with treatment strategies addressing anemia, splenomegaly, and symptom burden [2,6].

Although allogeneic stem cell transplantation (allo-SCT) remains the only therapy with curative potential, its application is often limited by significant transplant-related morbidity and mortality [7,8]. Beyond allo-SCT, treatments for myelofibrosis are mainly directed towards symptom control, though certain non-curative strategies can also improve overall survival and quality of life [[9], [10], [11]]. Therapeutic options include hydroxyurea and Janus kinase (JAK) 1/2 inhibitors, such as ruxolitinib, momelotinib, fedratinib, and pacritinib [1,4]. Of these, only ruxolitinib is currently available in Brazil. Other commonly used therapies encompass erythropoiesis-stimulating agents (ESAs), danazol, interferon, and immunomodulatory drugs, alongside interventions like splenectomy and radiotherapy [2,4]. Before the availability of JAK inhibitors, hydroxyurea was a frequently used first-line therapy worldwide [3]. Studies have demonstrated that hydroxyurea can lead to at least a 50% reduction in spleen size in approximately 30% of myelofibrosis patients, with a median duration of response of 13.2 months [12]. Another study reported clinical improvement or partial response in 28% of treated patients [13]. Ruxolitinib, an oral selective JAK1/2 inhibitor, demonstrated significant clinical benefit in the phase III COMFORT-I (versus placebo) and COMFORT-II (versus best available therapy) trials involving patients with intermediate-2 and high-risk myelofibrosis (IPSS) [11,14]. Notably, ruxolitinib was superior to best available therapy in achieving at least a 35% reduction in spleen volume at week 48 (28% versus 0%, respectively; p <0.001) [11]. Approximately 80% of responders maintained their response at a median follow-up of 12 months, with improvement in disease-related symptoms and quality of life [11].

The METER study was a comprehensive, multi-country, retrospective real-world study enrolling 998 myelofibrosis patients from 14 countries. This study described real-world treatment patterns, efficacy, clinical outcomes, and healthcare resource utilization in this patient population [15]. However, these features often vary across different countries and world regions. Such variation is commonly due to population characteristics, differential access to novel therapies, and local characteristics of healthcare systems. This is especially relevant in Brazil, where the healthcare system comprises both public and private sectors [16]. While the public sector provides universal coverage, it presents certain restrictions compared with the private sector, such as limitations in accessing services or drugs, particularly for cancer therapies [17]. Thus, the objective of the present analysis is to specifically evaluate the Brazilian cohort enrolled in the METER study, in order to investigate local treatment patterns and clinical outcomes for these patients.

## Methods

### *Study design and patient population*

This study is a non-interventional, retrospective chart review conducted as a subanalysis within the global METER study (NCT05444972) considering only patients enrolled in Brazil [15]. Data for the global study was collected between August 23, 2022 and November 14, 2023, and involved 66 participating centers from 14 countries. The Brazilian cohort consisted of 50 patients from two participating centers located in São Paulo (Hospital das Clínicas da Faculdade de Medicina da Universidade de São Paulo and Sociedade Beneficente Israelita Brasileira Hospital Albert Einstein). This subanalysis is based on the same eligibility criteria as in the original study. In brief, eligible patients were adults (≥18 years) diagnosed with primary or secondary myelofibrosis, irrespective of baseline risk, who received their first treatment on or after the local date of ruxolitinib approval and no later than December 31, 2021. Patients were treated according to routine clinical practice, with no influence from study participation. Patients who received treatment for myelofibrosis in a clinical trial were excluded. Data were collected on patient demographic and clinical characteristics, risk assessment, reported symptoms, blood-count analyses, treatment for myelofibrosis, hospitalization episodes, transfusion requirements, and survival status. Risk assessment data included the risk stratification method used at diagnosis (IPSS, DIPSS, DIPSS+, MIPSS) and the resulting risk category for each patient (low, intermediate-1, intermediate-2, and high risk). The index date was defined as the start date of the first documented myelofibrosis treatment, which could be initial medical therapy, allo-SCT, or splenectomy (collectively referred to as ‘procedure’), whichever occurred first. For each patient, existing individual-level data were collected from the medical charts by the study personnel at the participating centers until the last recorded contact or death, whichever came first. Data were inserted in the eCRF (Case Report Form) of the global METER study.

In Brazil, the METER study was approved by the Ethics Committee of the two participating centers. Written informed consent was obtained from patients whenever possible. Given the retrospective nature of the study, a waiver of informed consent was granted in specific circumstances. Situations considered as special circumstances were: research participants with no possibility of contact (no valid address or no success in telephone contact after at least three attempts), and deceased participants, in order to avoid possible embarrassment and emotional distress for the person legally responsible for the clinical data of the deceased participant.

### *Objectives and outcomes*

The primary objective of the current analysis was to describe real-world treatment patterns for myelofibrosis in the Brazilian cohort. This included characterizing patient demographic and clinical characteristics, time from myelofibrosis diagnosis to first-line therapy, choice and duration of initial and subsequent treatments, reasons for treatment changes or discontinuations, and utilization of treatment procedures. Secondary objectives included assessments of the number of transfusion events and the duration of all-cause hospital and intensive care unit (ICU) stays. Survival was the key treatment outcome analyzed, with other outcomes of interest being the time to treatment discontinuation/change for first- and second-line therapies, when applicable.

### Statistical analysis

All statistical analyses were performed using SAS, version 9.4 (SAS Institute Inc, Cary, NC) and R statistical software package. The analysis of the primary objective was descriptive. Continuous variables were presented as the number of observations, mean, standard deviation (SD), median, and lower (Q1) and upper quartiles (Q3). Categorical variables were summarized as the total number and percentages. Where appropriate, two-sided 95% confidence intervals (95% CI) for means and medians were computed. Time-to-event outcomes, such as time to treatment discontinuation/change and overall survival, were analyzed using the Kaplan-Meier method to estimate median times and their corresponding 95% CIs. Overall survival was analyzed both from the index date and from diagnosis of myelofibrosis and considered death from any cause; patients alive or loss to follow-up at the end of the observation period were censored at last contact. Hospital and ICU lengths of stay, as well as the number of transfusion events, were calculated and summarized for patients who experienced these events.

## Results

### *Baseline demographics and clinical characteristics*

Baseline patient characteristics are summarized in Table 1. A total of 50 patients from Brazil, 25 from each participating center, were included in the METER study and are analyzed here. Patients had been diagnosed according to the 2016 World Health Organization criteria. Of the 50 patients, 32 (64%) were male, the median age was 63.0 years (Q1-Q3: 57.5-70.0), and 29 (60.4%) were 65 years or younger. Regarding self-reported race, a substantial proportion was classified as unknown (n = 24; 48%), followed by White (n = 14; 28%) and Black or Afro-Brazilian (n = 12; 24%). Primary myelofibrosis was detected in 37 (74%) patients, while secondary myelofibrosis occurred in 13 individuals: post-polycythemia vera in seven (14%), and post-essential thrombocythemia in six (12%). The median time from myelofibrosis diagnosis to index date was 31 days (Q1-Q3: 1.0-131.5), with 19 (39.6%) occurring on the same day. At the time of diagnosis, bone marrow fibrosis grades were predominantly MF-3 (n = 21; 77.8%) and MF-2 (n = 5; 18.5%). Most patients were not transfusion-dependent at the index date (n = 29; 58%), although this information was unknown for a significant proportion of patients (n = 19; 38%).

**Table 1:** Demographics and patient characteristics.

Characteristic		n (%)
Sex	Male	32 (64.0)
	Female	18 (36.0)
Age at index date	Median age (Q1-Q3), years	63 (57.5-70.0)
Race*	White	14 (28.0)
	Black or Afro-Brazilian	12 (24.0)
	Asian	0
	Unknown	24 (48.0)
Ethnicity	Hispanic or Latino	25 (50.0)
	Not Hispanic or Latino	11 (22.0)
	Unknown	14 (28.0)
Type of myelofibrosis	Primary	37 (74.0)
	Secondary	13 (26.0)
Bone marrow fibrosis at diagnosis	MF-0	0
	MF-1	0
	MF-2	5 (18.5)
	MF-3	21 (77.8)
	Unknown	1 (3.7)
	Missing^⁎⁎^	23
Hematologic profile at baseline	Hemoglobin (g/dL), median (Q1-Q3)	9.75 (8.40-11.80)
	Platelet count (x 10^9^/L), median (Q1-Q3)	302.0 (147.0-444.0)
Transfusion dependency at index date	Yes	2 (4.0)
	No	29 (58.0)
	Unknown	19 (38.0)

*Information about race was obtained from medical charts and self-reported by patients.

^⁎⁎^Missing category may include patients who did not complete testing/classifications or had missing information for corresponding questions. Q1-Q3: lower and upper quartiles.

The predominant risk classification method was DIPSS (n = 26; 60.5%), followed by DIPSS+ (n = 8; 18.6%) (Table 2). At diagnosis, nine (20.9%) individuals had high risk disease, 14 (32.6%) intermediate-2, 12 (27.9%) intermediate-1, and eight (18.6%) low risk. Molecular evaluation data was missing for approximately 64% of patients. Among the 18 patients with available data, 50% (n = 9) presented a high-risk mutation at diagnosis.

**Table 2:** Risk classification at diagnosis.

Risk classification		n (%)
Risk classification method at diagnosis	IPSS	6 (14.0)
	DIPSS	26 (60.5)
	DIPSS+	8 (18.6)
	MIPSS	2 (4.7)
	Unknown	1 (2.3)
	Missing*	7
Risk classification at diagnosis	Low	8 (18.6)
	Intermediate-1	12 (27.9)
	Intermediate-2	14 (32.6)
	High	9 (20.9)
	Missing*	7
High molecular risk mutation at diagnosis	Yes	9 (50.0)
	No	8 (44.4)
	Unknown	1 (5.6)
	Missing*	32

DIPSS: Dynamic International Prognostic Scoring System; DIPSS+: Dynamic International Prognostic Scoring System Plus; IPSS: International Prognostic Scoring System; MIPSS: Mutation-enhanced International Prognostic Scoring System.

*Missing category may include patients who did not complete testing/classifications or had missing information for corresponding questions.

The most prevalent comorbidities at myelofibrosis onset were malignancy (n = 19 of 39 patients with available data; 48.7%), cardiovascular disease (n = 19 of 40; 47.5%), and endocrine disease (n = 16 of 39; 41.0%). The median number of symptoms at baseline was two (Q1-Q3: 1-3). At weeks 24, 52, and 104, the median number of symptoms reduced to one (Q1-Q3: 0-2), one (Q1-Q3: 0-2), and zero (Q1-Q3: 0-2), respectively.

### *Initial treatment*

Among the 50 patients, 32 (64%) received hydroxyurea as initial treatment. Ruxolitinib was the second most common option for first-line therapy, administered to 12 (24%) patients, followed by ESAs in 11 (22%). The most common supportive care medications in the initial setting included bisphosphonates (12%), antiemetics (12%), and nutritional support (8%).

Table 3 displays the frequency and reasons for treatment discontinuation or change by line of therapy. A total of 23 patients (46%) had discontinuation or change of initial therapy, with a median overall treatment duration of 29.9 months (95% CI: 21.5-not estimable [NE]). The main reasons for discontinuation or change in first line were inadequate response (n = 11/23; 47.8%), death (n = 6/23; 26.1%), and disease progression (n = 3/23; 13.0%).

**Table 3:** Treatment discontinuation or change by treatment lines.

	Initial treatment (N = 50) n (%)	Treatment 2 (N = 12) n (%)	Treatment 3 (N = 4) n (%)	Treatment 4 (N = 1) n (%)
Main treatment ongoing	27 (54.0)	5 (41.7)	2 (50.0)	1 (100)
Main treatment discontinued or changed	23 (46.0)	7 (58.3)	2 (50.0)	0
Reason for discontinuation or change				
Inadequate response*	11 (47.8)	5 (71.4)	1 (50.0)	0
Disease progression*	3 (13.0)	0	0	0
Completed planned treatment	0	1 (14.3)	0	0
Decline in performance status	1 (4.3)	0	0	0
Death	6 (26.1)	0	1 (50.0)	0
Physician preference	1 (4.3)	0	0	0
Other	1 (4.3)	1 (14.3)	0	0

Analysis set includes all patients who received at least one dose of the first treatment. For a patient with multiple treatment lines, the reason for each drug discontinuation/change is counted. One treatment line per patient has only one discontinuation reason.

*The European Leukemia Net (ELN) criteria were used for response evaluation. Information regarding inadequate response and disease progression recorded as the reason for discontinuation or change was retrieved from medical charts, according to the physicians/investigator´s assessment at the respective time.

### *Second-line treatment*

Twelve patients received second-line therapy, with the most frequent being ruxolitinib in six individuals (50%), and chemotherapy in three (25%). Hydroxyurea and ESAs were the therapeutic choice for two (16.7%) patients each. The most frequently required supportive therapies in this setting were antiemetics (33.3%) and nutritional support (16.7%). Seven patients (58.3%) starting second-line therapy experienced treatment discontinuation or change, with a median overall treatment duration of four months (95% CI: 0.7-NE). The main reason for discontinuation or change was inadequate response (n = 5/7; 71.4%) (Table 3).

### *Procedural intervention*

Nine patients underwent a myelofibrosis procedure, with a median time from index date to procedure of 367 days (Q1-Q3: 207-951). The most common procedures were allo-SCT in six individuals (median time 251 days), splenectomy in two (659 days), and radiotherapy in one patient (688 days).

### *Health care resource utilization and complications*

Of the patients who were eventually hospitalized after the index date (n = 29), the median duration of hospitalization was 3.0 days (Q1-Q3: 0.0-28.0), and the mean number of days in ICU was 1.3 (SD: 4.31). Among those who required transfusion after the index date (n = 20), the mean number of transfusion events was 4.4 (SD: 10.37).

Six patients developed acute myeloid leukemia (AML) transformation. The cumulative incidence from start date of initial treatment to AML transformation increased over time, with 4.1% at week 24, 9.0% at week 104, and 12.8% at week 156.

### *Overall survival*

Deaths occurred in 19 (38%) patients during the follow-up period. The median overall survival from index date was 45.5 months (95% CI: 28.5-NE), with 24-week, 52-week, 104-week, and 156-week survival rates of 98%, 87.3%, 74.7%, and 57.7%, respectively. The median overall survival from myelofibrosis diagnosis was 142.3 months (95% CI: 30.8-NE), with 24-week and 156-week survival estimates of 100% and 62.9%, respectively.

## Discussion

This report presents a subgroup analysis of the Brazilian cohort, comprised of 50 patients of two participating centers, within the multi-country METER study, which provided a real-world assessment of 998 patients with myelofibrosis across 14 countries [15]. Recognizing that country-specific factors such as population characteristics, treatment access, and healthcare systems can significantly influence disease management, this analysis aimed to delineate the unique aspects of myelofibrosis care in Brazil.

Except for race, baseline patient characteristics were generally comparable to those from the overall METER study population, which includes the Brazilian cohort. Of note, primary myelofibrosis was slightly more frequent in Brazil (74.0%) than overall (65.9%). Although the Brazilian cohort showed a lower proportion of transfusion dependence at the index date, this analysis was limited by a substantially larger frequency of missing data (38% vs. 7.4%). Notwithstanding the high frequency of data missingness, similar rates of high-risk mutations (approximately 50%) and a comparable burden of higher-risk disease (intermediate-2 and high risk combined) were noted between the Brazilian and overall cohorts.

The high rate of missing molecular data in the Brazilian cohort underscores a frequent occurrence in this country, namely a significant barrier to state-of-the-art diagnostic methods in oncology [18,19]. As highlighted by previous research, limited access to mutational profiling by most Brazilian centers hinders accurate risk stratification and may reduce the identification of patients who would benefit most from early consideration of allo-SCT, thus hampering optimal myelofibrosis management [8]. Moreover, it is possible that the true proportion of high-risk patients in Brazil may be underestimated if data missingness is somehow associated with disease risk. For example, a study from a Brazilian tertiary center reported a higher prevalence of high-risk mutations in their cohort [20], although this has not been demonstrated by other series [21]. Furthermore, database surveys of Brazilian patients with myelofibrosis indicated potential issues with diagnostic completeness, including access to necessary laboratory tools [22,23].

Notably, hydroxyurea was the most frequent initial treatment in Brazil (64%), contrasting with the overall METER study and a US-based real-world study, in which ruxolitinib was the most commonly prescribed first-line therapy [15,24]. In Brazil, ruxolitinib was the most common second-line treatment (50%). In this country, access to novel therapies is hampered by lack of reimbursement in public institutions and is often obtained through judicial means [25]. Therefore, the observed preference for hydroxyurea as initial treatment in Brazil likely reflects existing challenges in accessing JAK inhibitors, which may be linked to the distinct healthcare landscape in the country, characterized by disparities between the public and private systems [8,26]. Access to state-of-the-art therapies for myelofibrosis remains a challenge for the public healthcare system, with restricted availability of drugs such as JAK inhibitors [8]. In the private healthcare system, although there may be some degree of access restrictions, reimbursement of drugs and procedures is generally broader [26].

The rate of discontinuation or change of initial therapy and the median duration of first-line treatment were similar between the Brazilian and overall METER cohorts. However, only 24% of patients in the Brazilian group progressed to second-line therapy (accounting for 52% of those who discontinued or changed the initial therapy). In contrast, in the overall METER analysis and the US-based real-world study, about 40% of individuals received two or more lines of treatment (representing 75% of patients who discontinued or changed the initial therapy in the overall METER study) [15,24]. The lower rate of progression to second-line therapy in the Brazilian cohort could be multifactorial. It might reflect earlier mortality, limited access to subsequent therapies, or different clinical decision-making processes. Additionally, a notable finding was the higher rate of death as a reason for discontinuation or change of initial treatment in Brazil (26.1%), in comparison with the overall METER cohort (6.1%). This higher rate raises concerns about potential delays in diagnosis, referral to specialized centers, and access to optimal therapies in the Brazilian setting [26]. A retrospective study from Brazilian hematology centers reported a substantial delay from symptom onset to diagnosis [23], which could contribute to patients presenting at more advanced stages, thus being less fit or having a worse prognosis upon initiating treatment. On the other hand, the higher rate of death as a reason for first-line treatment discontinuation may have alternative explanations, including those related to subjectivity in ascertainment of this and competing reasons.

Importantly, the current study shows that the median overall survival from the index date was considerably shorter for the Brazilian subgroup (45.5 months) than for the overall METER cohort [15]. The key potential explanations for the observed survival difference are biological factors and treatment patterns. Given that disease risk did not differ considerably between the two cohorts, different treatment patterns are a plausible explanation for the survival difference. The 5-year data from COMFORT-1 demonstrated prolonged overall survival with ruxolitinib versus placebo (not reached versus 200 weeks; HR: 0.69; 95% CI: 0.50-0.96; p = 0.025), despite crossover [10]. A post-hoc pooled analysis from COMFORT-I and COMFORT-II also reported survival benefit in favor of ruxolitinib (HR = 0.65; 95% CI: 0.46-0.90; p = 0.01) [9]. Real-world studies like ERNEST have shown improved survival in patients treated with ruxolitinib versus hydroxyurea (6.7 versus 5.1 years, respectively; p = 0.001), even after multivariate analysis [27]. While the Brazilian cohort of the JUMP trial showed positive outcomes with ruxolitinib [28], the limited use of JAK inhibitors as initial therapy in the Brazilian METER cohort may have contributed to the poorer survival observed in this subanalysis.

The present study, however, has some limitations that warrant consideration. The retrospective nature of this chart review and the relatively small sample size of the Brazilian cohort potentially impose biases and limit the required precision for more granular analyses and indirect comparisons. The significant amount of missing data hampers a complete understanding of the patient characteristics and outcomes. Furthermore, a limitation of this study is the lack of specific analyses assessing the impact of healthcare financing source (public versus private) a well-recognized area of disparity in Brazil [8,26] in respect to patient characteristics, treatment patterns, and outcomes.

## Conclusion

This subanalysis of the METER study highlights distinct treatment patterns for myelofibrosis in Brazil in comparison with the overall analysis, notably the prevalent use of hydroxyurea as first-line therapy and the infrequency of use of JAK inhibitors. The findings also underscore challenges related to access to molecular diagnostics and potentially to advanced therapies, which may contribute to the observed shorter overall survival. Future research should focus on assessing healthcare access and improving treatment availability in Brazil.

## Funding

This study was funded by AbbVie, which contributed to the study design and reviewed the manuscript for accuracy, without involvement in data collection, analysis, interpretation, or writing.

## Data availability

The data that support the findings of this study are available from the corresponding author upon reasonable request.

## Conflicts of interest

Fernanda Seguro does not have conflicts to declare. Ingrid Pereira and Maria Duran are employed with AbbVie. Nelson Hamerschlak have participation on clinical trials and expanded access program of ABBVIE and GSK.
